# Combination of Peptide YY_3–36_ with GLP-1_7–36 amide_ Causes an Increase in First-Phase Insulin Secretion after IV Glucose

**DOI:** 10.1210/jc.2014-2143

**Published:** 2014-08-21

**Authors:** Tricia M. Tan, Victoria Salem, Rachel C. Troke, Ali Alsafi, Benjamin C. T. Field, Akila De Silva, Shivani Misra, Kevin C. R. Baynes, Mandy Donaldson, James Minnion, Mohammad A. Ghatei, Ian F. Godsland, Stephen R. Bloom

**Affiliations:** Section of Investigative Medicine (T.M.T., V.S., R.C.T., A.A., B.C.T.F., A.D.S., S.M., K.C.R.B., M.D., J.M., M.A.G., S.R.B.), and Department of Endocrinology and Metabolism (I.F.G.), Imperial College London, London W12 0NN, United Kingdom

## Abstract

**Context:**

The combination of peptide YY (PYY) and glucagon-like peptide-1 (GLP-1) has been proposed as a potential treatment for diabetes and obesity. However, the combined effects of these hormones, PYY_3–36_ and GLP-1_7–36 amide_, on glucose homeostasis are unknown.

**Objective:**

This study sought to investigate the acute effects of PYY_3–36_ and GLP-1_7–36 amide_, individually and in combination, on insulin secretion and sensitivity.

**Setting and Design:**

Using a frequently sampled iv glucose tolerance test (FSIVGTT) and minimal modeling, this study measured the effects of PYY_3–36_ alone, GLP-1_7–36 amide_ alone, and a combination of PYY_3–36_ and GLP-1_7–36 amide_ on acute insulin response to glucose (AIRg) and insulin sensitivity index (S_I_) in 14 overweight human volunteers, studied in a clinical research facility.

**Results:**

PYY_3–36_ alone caused a small but nonsignificant increase in AIRg. GLP-1_7–36 amide_ alone and the combination of PYY_3–36_ and GLP-1_7–36 amide_ did increase AIRg significantly. No significant differences in S_I_ were observed with any intervention.

**Conclusions:**

PYY_3–36_ lacks any significant acute effects on first-phase insulin secretion or S_I_ when tested using an FSIVGTT. Both GLP-1_7–36 amide_ alone and the combination of PYY_3–36_ and GLP-1_7–36 amide_ increase first-phase insulin secretion. There does not seem to be any additive or synergistic effect between PYY_3–36_ and GLP-1_7–36 amide_ on first-phase insulin secretion. Neither hormone alone nor the combination had any significant effects on S_I_.

The obesity pandemic has become a priority global health concern. By 2015, the World Health Organization predicts that four billion adults will be overweight and more than 700 million will be obese. This has predictably resulted in an increase in prevalence of the comorbidities of obesity, including type 2 diabetes, cardiovascular disease, hypertension, cancer, and obstructive sleep apnea, all resulting in a reduced life expectancy (1, 2). Bariatric surgery, for example the Roux-en-Y gastric bypass, is currently the most effective treatment, leading to a sustained 25–30% weight loss (3–5). It is well documented that gastric bypass surgery also induces a rapid and prolonged improvement in glucose levels, with greater than 40% of operated subjects achieving a complete remission of their diabetes (6). At present, the mechanisms for the favorable changes in body weight and glycemia are unclear, although alterations in peptide gut hormone secretion—in particular elevated postprandial levels of peptide YY (PYY) and glucagon-like peptide-1 (GLP-1)—are thought to play an important role (7, 8). As a result, analogs of PYY and GLP-1 are being developed as treatments for obesity and diabetes, although the doses that can be given are limited by adverse effects, principally nausea (9, 10). Low-dose combinations of PYY and GLP-1 represent an attractive route to achieving better weight-lowering efficacy without nausea: we have previously shown that PYY_3–36_ coinfused with GLP-1_7–36 amide_ reduces appetite and food intake in an additive fashion without adverse effects (11, 12).

GLP-1 is well established as an incretin hormone with insulinotropic effects (13). In contrast, the PP-fold (pancreatic polypeptide-fold) peptides neuropeptide Y (NPY) and PYY have differing effects on insulin secretion. NPY, acting at the neuropeptide Y1 receptor, inhibits insulin release from islets (14, 15), and sympathetic nerve terminals on pancreatic islets release both NPY and norepinephrine to produce inhibition of insulin secretion (16). Consistent with this, Y1 receptor knockout mice exhibit a basal hyperinsulinemia (17). PYY_1–36_, the full-length version of PYY that is able to activate Y1 receptors, similarly has insulinostatic effects in rodents and dogs (18–21), although it seems to increase insulin secretion after an ad libitum meal when infused into humans at 1.6 pmol/kg/min (22). Unlike NPY and PYY_1–36_, relatively little is known about the effects of PYY_3–36_ on glucose metabolism. PYY_3–36_ is considerably less active at the Y1 receptor but fully active at the neuropeptide Y2 receptor. Given that the Y2 receptor has been shown to act as a presynaptic auto-inhibitor of sympathetic transmission (23), Y2 activation might not affect or could even cause disinhibition of insulin release. In animal studies, administration of PYY_3–36_ was associated with increased glucose disposal under hyperinsulinaemic conditions, ie, an increase in insulin sensitivity (24). In humans, Sloth et al (22) reported that an acute iv infusion of PYY_3–36_ (at a dose of 0.2 pmol/kg/min, achieving mean levels of 76 ± 23 pmol/L) was able to increase the postprandial insulin response to an ad libitum meal, as judged by area under the curve (AUC) for insulin concentration.

If low-dose combinations of PYY_3–36_ and GLP-1_7–36 amide_ are to be used as future treatments for obesity and/or diabetes, it is important to establish their effects on glucose homeostasis. We therefore decided to investigate the effects of PYY_3–36_ and GLP-1_7–36 amide_, individually and in combination, on insulin secretion and sensitivity in fasted overweight humans.

## Materials and Methods

### Peptides

PYY_3–36_ and GLP-1_7–36 amide_ were purchased from Bachem. Following initial high-fidelity synthesis, the peptide hormones underwent purification by high-resolution, high-performance liquid chromatography. Peptide compositions and purity were verified by quantitative amino acid analysis.

Sterile 0.9% (w/v) saline was purchased from Bayer. Using an aseptic technique in a laminar flow cabinet, PYY_3–36_ and GLP-1_7–36 amide_ were separately dissolved in 0.9% saline, aliquoted into vials, and freeze dried. Representative PYY_3–36_ and GLP-1_7–36 amide_ vials were sterile after culture for 7 days (Department of Microbiology, Hammersmith Hospital, London, United Kingdom), and endotoxin levels as measured by the Limulus Amoebocyte Lysate test (Associates of Cape Cod) were within the safe range for human infusion. Further representative vials of both PYY_3–36_ and GLP-1_7–36 amide_ were randomly selected and sent for amino acid analysis by Alta Bioscience to calculate the actual peptide content of the vials. The bioactivity of the peptides was verified by measuring the suppression of food intake over 24 hours when injected sc into C57/BL6 mice (12), and by receptor-binding affinity assays using membranes prepared from HEK293 cells overexpressing recombinant human Y2 or GLP-1 receptor (25).

### Subjects

Fourteen overweight and obese volunteers, 11 men and 3 women, of mean age 34.5 ± 2.7 years (range, 21–50 years), and mean body mass index 30.1 ± 0.9 kg/m^2^ (range, 26.8–35.9 kg/m^2^), were recruited by advertisement. All volunteers underwent a standardized 75 g oral glucose tolerance test to exclude both diabetes and impaired glucose tolerance, to reduce variability in insulin secretion and sensitivity. Inclusion criteria were: age, 18 years and older; sex, male or female; body mass index, 25–40 kg/m^2^; and nonsmokers with stable weight for at least 3 months. Exclusion criteria were: diabetes mellitus or impaired glucose tolerance according to World Health Organization 2006 and 2011 criteria; history of alcoholism or substance abuse; and history of any major illness or use of any medications including over-the-counter products, which, in the opinion of the investigator, would either interfere with the study or potentially cause harm to the volunteer. Women who were pregnant, breastfeeding, or unable to maintain adequate contraception for the duration of the study and for 1 month afterward were also excluded.

All volunteers were screened and determined to be in normal health by medical history, physical examination, 12-lead electrocardiogram, and routine biochemistry and hematology. Women of child-bearing age were advised to avoid pregnancy during the study and for 1 month after completion. The study was approved by the Hammersmith & Queen Charlotte's Research Ethics Committee (Reference No. 09/H0707/77). All volunteers gave written informed consent, and the study was planned and performed in accordance with the Declaration of Helsinki.

### Study protocol

Each volunteer attended five study visits. The first visit was to acclimatize the volunteer to the clinical environment and to experimental procedures. This acclimatization visit was run in identical fashion to subsequent, randomized, single-blinded visits, except that the infusion always consisted only of vehicle. Data from the acclimatization visit was not included in the analysis. The subsequent four visits followed a randomized, single-blind, placebo-controlled crossover design comparing four different infusions: 1) Vehicle alone (Gelofusine; B. Braun Medical); 2) PYY_3–36_ alone (0.15 pmol/kg/min); 3) GLP-1_7–36 amide_ alone (0.2 pmol/kg/min); 4) PYY_3–36_ + GLP-1_7–36 amide_ together (0.15 pmol/kg/min and 0.2 pmol/kg/min, respectively). The infused doses of the peptide hormones were selected after a preliminary dose-finding phase to achieve plasma concentrations of PYY_3–36_ at 80–120 pmol/L, a level that has previously been shown to increase postprandial insulin AUC values after an ad libitum meal (22). For GLP-1_7–36 amide_, we aimed to achieve 100–140 pmol/L, a level that has previously been shown to increase insulin secretion rate in response to a graded glucose infusion (26). Randomization was carried out by an independent clinician not otherwise involved in the study.

To limit adsorption of peptide to the infusion apparatus, Gelofusine was used as the vehicle for all peptide infusions to dissolve the contents of the randomized vials of peptide and to prime all syringes and infusion lines (27). Each peptide was drawn up under sterile conditions in a separate 50-mL syringe, and to enable the use of two different infusion rates, was delivered by separate syringe drivers (Graseby 3100, SIMS Graseby or Asena GH Mk III, Alaris Medical Systems). Thus, on a visit when the volunteer received only one peptide, the second syringe delivered vehicle only, set at the delivery rate calculated for the other hormone. Study visits for each volunteer were at least 3 days apart to allow for washout of peptides and peptide effects. During the 24-hour period prior to each study visit, volunteers refrained from strenuous exercise and alcohol consumption. They fasted from 2200 h the night before the study, drinking only water. On the morning of each study visit, volunteers attended a dedicated Clinical Investigation Unit at the Hammersmith Hospital. Female volunteers underwent a urine β-hCG test to exclude pregnancy before the peptide infusion was started. Two cannulae were inserted into the volunteer's peripheral veins. One cannula was used for sampling and the other one was used to administer peptide infusion and iv glucose bolus (via a multiport connector). The infusion containing the peptide hormone(s) was started at 0 minutes. For evaluation of the acute insulin response to glucose (AIRg) and insulin sensitivity index (S_I_), a frequently sampled iv glucose tolerance test (FSIVGTT) was performed at +60 minutes with an iv glucose bolus of 0.3 g/kg administered manually over 2 minutes (28). Augmentation of iv glucose tolerance test plasma insulin concentrations by tolbutamide or insulin injection was not undertaken because volunteers were normoglycaemic and insulin release was, in any case, likely to be amplified by the GLP-1 infusions. The peptide infusion was stopped at +240 minutes. Volunteers completed a series of visual analog scales that rated hunger, satiety, prospective food consumption, and nausea throughout the study. These consisted of 100-mm lines with text expressing the most positive and the most negative rating for each variable anchored at either end (29). Pulse and blood pressure were regularly monitored.

Blood samples were taken for glucose into fluoride oxalate tubes, and insulin into plain serum tubes (Becton, Dickinson) at −30, 0, 20, 40, 60, 62, 63, 64, 65, 66, 68, 70, 72, 74, 78, 80, 82, 85, 90, 100, 110, 130, 160, 200, and 240 minutes. Larger samples were taken at 0, 20, 40, 60, 80, 100, 160, and 240 minutes for plasma gut hormone analysis in lithium heparin coated tubes (International Scientific Supplies) containing 2000 kallikrein inhibitor units (0.2 ml) aprotinin (Trasylol, Bayer Schering Pharma). The insulin samples were allowed to clot for 10 minutes at room temperature, after which they were centrifuged and separated and stored at −20°C until analysis. All other samples underwent immediate centrifugation for 10 minutes, 4000 rpm at 4°C, after which plasma was promptly separated and stored at –20°C until analysis.

### Plasma gut hormone assays

All samples were assayed in duplicate and within a single assay to eliminate interassay variation. Serum insulin was assayed using the Siemens Immulite 2000 immunoassay, which is a solid-phase, two-site chemiluminescent immunoassay with an analytical range of 2–300 mIU/L and an intra-assay coefficient of variation of 3.3–5.5%. Plasma glucose was assayed using an Abbott Architect automated analyzer using a hexokinase-glucose-6-phosphate dehydrogenase method. The analytical range was 0.278–44.4 mmol/L with an intra-assay coefficient of variation of 0.65–1.98% and an interassay coefficient of variation of 0.84–0.93%. Plasma total PYY and amidated GLP-1 were measured using established in-house RIAs (30, 31). The PYY assay's functional detection limit was 16.8 pmol/L (95% confidence interval [CI] 14.4–19.3) with an intra-assay coefficient of variation of 7.4%. The GLP-1 assay's functional detection limit was 13.4 pmol/L (95% CI, 12.5–14.2) with an intra-assay coefficient of variation of 3.1%.

### Data analysis

Data are expressed as mean ± SEM except where noted. Statistical analysis was carried out using Prism 5.0 (GraphPad Software). The acute plasma insulin concentration response to glucose (AIRg, 0–10 min), a sensitive index of beta cell function and first-phase insulin response (32), was calculated as the area under the FSIVGTT insulin concentration profile (AUC) from 0–10 minutes following glucose administration, calculated using the trapezoid rule (33). The insulin sensitivity index S_I_, a measure of the ability of insulin to enhance glucose disposal, was determined from FSIVGTT glucose and insulin concentrations using the minimal model of glucose disappearance (34) implemented as previously described (35). The FSIVGTT-derived measures, AIRg and S_I_ provide the so-called disposition index (DI), calculated as S_I_ × AIRg (36). This widely used, dimensionless measure of beta cell function quantifies beta cell adaptation to variation in S_I_ according to the hyperbolic relationship between insulin resistance and insulin secretion.

## Results

**Figure 1.**
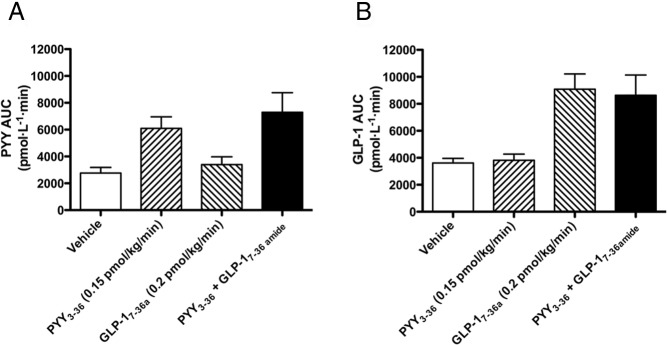
A, PYY; and B, GLP-1 exposure during the FSIVGTT. Integrated area AUC for 0–100 min, from the start of the infusion to the end of the intensive minimal modeling period, is plotted on the y-axis. The x-axis indicates infusion given. Mean ± SEM plotted. Baseline plasma PYY levels (at 0 min) were vehicle, 47.7 ± 8.7 pmol/L; PYY_3–36_, 45.8 ± 8.1 pmol/L; GLP-1_7–36 amide_, 34.1 ± 5.3 pmol/L; PYY_3–36_ + GLP-1_7–36 amide_, 52.2 ± 10.9 pmol/L). End-infusion (+240 min: steady state) levels were vehicle, 26.7 ± 15.8 pmol/L; PYY_3–36_, 113.5 ± 13.7 pmol/L; GLP-1_7–36 amide_, 21.3 ± 13.9 pmol/L; PYY_3–36_ + GLP-1_7–36 amide_, 97.8 ± 37.2 pmol/L. To estimate the exposure of volunteers to PYY_3–36_ from 0–100 min, the respective AUC for each infusion arm was calculated as follows: vehicle, 2766 ± 423.7 pmol · L^−1^ · min; PYY_3–36_, 6091 ± 861.2 pmol · L^−1^ · min; GLP-1_7–36 amide_, 3395 ± 575.9 pmol · L^−1^ · min; PYY_3–36_ + GLP-1_7–36 amide_, 7297 ± 1460 pmol · L^−1^ · min. Baseline plasma GLP-1 levels (at 0 min) across different infusion arms were vehicle, 43.7 ± 6.2 pmol/L; PYY_3–36_, 43.8 ± 7.7 pmol/L; GLP-1_7–36 amide_, 55.6 ± 9.7 pmol/L; PYY_3–36_ + GLP-1_7–36 amide_, 52.6 ± 15.2 pmol/L). End-infusion (+240 min: steady state) levels were vehicle, 44.0 ± 8.5 pmol/L; PYY_3–36_, 33.4 ± 2.6 pmol/L; GLP-1_7–36 amide_, 142.2 ± 22.3 pmol/L; PYY_3–36_ + GLP-1_7–36 amide_, 140.4 ± 22.0 pmol/L. To estimate the exposure of volunteers to GLP-1_7–36 amide_ from 0–100 min, the respective AUC for each infusion arm was calculated as follows: vehicle, 3614 ± 344.2 pmol · L^−1^ · min; PYY_3–36_, 3813 ± 458.7 pmol · L^−1^ · min; GLP-1_7–36 amide_, 9084 ± 1134 pmol · L^−1^ · min; PYY_3–36_ + GLP-1_7–36 amide_, 8639 ± 1495 pmol · L^−1^ · min.

Table 1 summarizes the average plasma hormone concentrations achieved in each of the study arms. PYY exposures were similar between the two arms that included PYY_3–36_ in the infusion (Figure 1A), as were GLP-1 exposures comparing the arms that included GLP-1_7–36 amide_ in the infusion (Figure 1B). There were no variations in pulse and blood pressure across infusions and analysis of visual analog scale scores revealed no nausea in response to the gut hormone infusions nor any differences in subjective ratings of hunger or pleasantness to eat between any of the interventions (data not shown).

**Table 1.** Summary of PYY and GLP-1 Plasma Concentrations (in pmol/L) and Area Under The Curve Values (pmol · L^−1^ · min) for Each of the Infusion Arms: Vehicle, PYY_3–36_, GLP-1_7–36 amide_, and Combination

Infusion Arm	Baseline Plasma Concentration, pmol/L	End-Infusion Plasma Concentration, pmol/L	AUC Plasma Concentrations Over Infusion Period, pmol · L^−1^ · min
PYY levels			
Vehicle	47.7 ± 8.7	26.7 ± 15.8	2766 ± 423.7
PYY	45.8 ± 8.1	113.5 ± 13.7	6091 ± 861.2
GLP-1	34.1 ± 5.3	21.3 ± 13.9	3395 ± 575.9
Combination	52.2 ± 10.9	97.8 ± 37.2	7297 ± 1460
GLP-1 levels			
Vehicle	43.7 ± 6.2	44.0 ± 8.5	3614 ± 344.2
PYY	43.8 ± 7.7	33.4 ± 2.6	3813 ± 458.7
GLP-1	55.6 ± 9.7	142.2 ± 22.3	9084 ± 1134
Combination	52.6 ± 15.2	140.4 ± 22.0	8639 ± 1495

**Figure 2.**
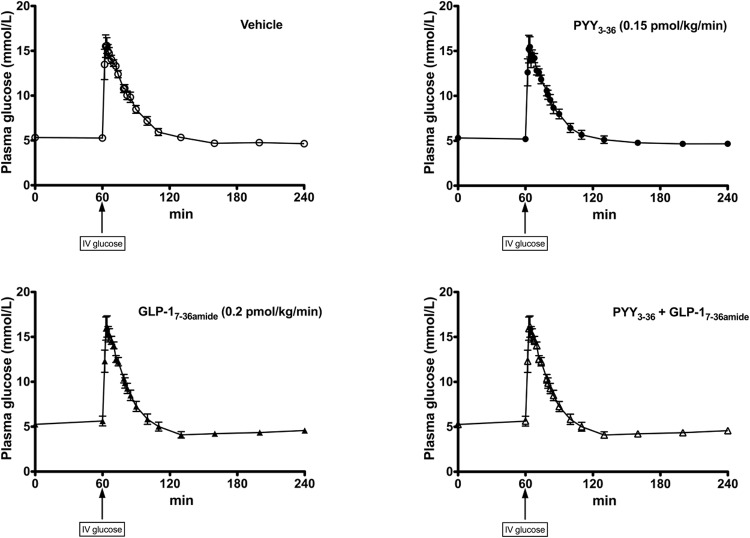
Plasma glucose levels during the FSIVGTT. Y-axis shows plasma glucose levels (mmol/L). X-axis shows time (min). iv glucose bolus (0.3 g/kg) given at 60 min. Mean ± SEM plotted. Open circles, dashed line placebo infusion arm; closed circles, solid line: PYY_3–36_ infusion (0.15 pmol/kg/min). Closed triangles, solid line: GLP-1_7–36 amide_ infusion (0.2 pmol/kg/min). Open triangles, solid line: combined PYY_3–36_ + GLP-1_7–36 amide_ infusion. Fasting glucose values for vehicle, 5.3 ± 0.1 mmol/L; PYY_3–36_, 5.3 ± 0.2 mmol/L; GLP-1_7–36 amide_, 5.3 ± 0.1 mmol/L; combined PYY_3–36_ + GLP-1_7–36 amide_, 5.4 ± 0.1 mmol/L.

Fasting glucose levels were very similar between all infusion arms (Figure 2). With the administration of the iv glucose bolus, glucose levels peaked at 15.5–16.2 mmol/L (64 minutes) and decreased back to baseline by 110 minutes. In no case did any volunteer experience a biochemical or symptomatic hypoglycemia as a result of the endogenous insulin release in response to the large iv glucose bolus.

**Figure 3.**
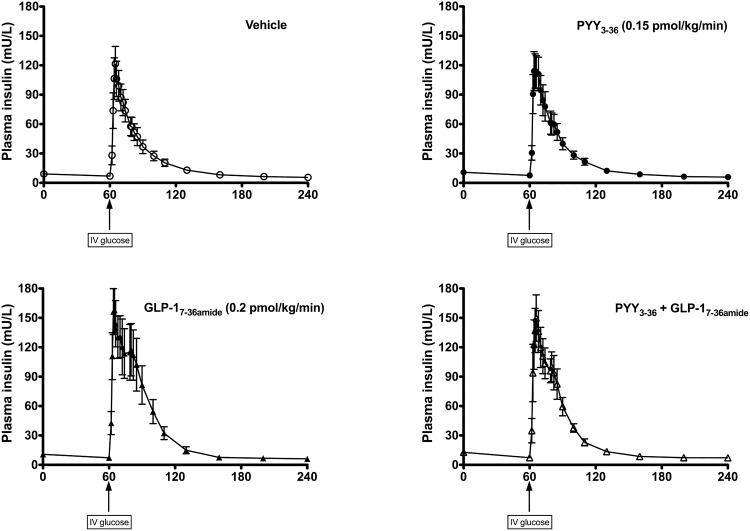
Plasma insulin levels during the FSIVGTT. Y-axis shows insulin levels (mU/L). X-axis shows time (min). iv glucose bolus (0.3 g/kg) given at 60 min. Mean ± SEM plotted. Open circles, dashed line: placebo infusion arm. Closed circles, solid line: PYY_3–36_ infusion (0.15 pmol/kg/min). Closed triangles, solid line: GLP-1_7–36 amide_ infusion (0.2 pmol/kg/min). Open triangles, solid line: combined PYY_3–36_ + GLP-1_7–36 amide_ infusion.

**Figure 4.**
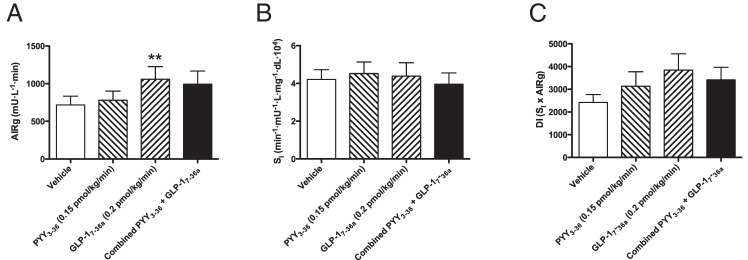
A, AIRg response to iv glucose. Means plotted ± SEM, one-way repeated measures ANOVA (*P* = .0046). AIRg means for vehicle 718.1 ± 115.7 mU · L^−1^ · min; GLP-1_7–36 amide_ infusion, 1060 ± 167.6 mU · L^−1^ · min; PYY_3–36_, 778.8 ± 122.4 mU · L^−1^ · min; combined PYY_3–36_ + GLP-1_7–36 amide_, 993.5 ± 173.3 mU · L^−1^ · min. *, = *P* < .05 and **, *P* < .01 for comparison of combined and GLP-1 (respectively) to vehicle for AIRg by Bonferroni's multiple correction test. B, S_I_. Means plotted ± SEM, one-way repeated measures ANOVA (*P* = .7929). S_I_ for vehicle, 4.21 ± 0.51 min^−1^ · mU^−1^ · L · mg^−1^ · dL · 10^4^; PYY_3–36_, 4.52 ± 0.61 min^−1^ · mU^−1^ · L · mg^−1^ · dL · 10^4^; GLP-1_7–36 amide_, 4.39 ± 0.71 min^−1^ · mU^−1^ · L · mg^−1^ · dL · 10^4^; combined PYY_3–36_ + GLP-1_7–36 amide_, 3.96 ± 0.59 min^−1^ · mU^−1^ · L · mg^−1^ · dL · 10^4^. C, DI. Means plotted ± SEM, one-way repeated measures ANOVA (*P* = .07). DI for vehicle, 2417 ± 350; PYY_3–36_, 3131 ± 638; GLP-1_7–36 amide_, 3844 ± 717; combined PYY_3–36_ + GLP-1_7–36 amide_, 3414 ± 554.

The insulin response to the iv glucose bolus is shown in Figure 3. Infusion of GLP-1_7–36 amide_, either alone or in combination with PYY_3–36_, augmented the insulin-secretory response following the iv glucose bolus compared with either vehicle or PYY alone. In line with this, the AIRg during each infusion showed a significant difference in means (*P* = .005; Figure 4A). No significant difference was detected on post-hoc testing between vehicle and PYY_3–36_ (mean difference in AIRg, 60.71 mU · L^−1^ · min, 95% CI for difference, −210.2–331.7). A significant difference was detected between vehicle and GLP-1_7–36 amide_ (*P* < .01: mean difference in AIRg, 341.7 mU · L^−1^ · min; 95% CI for difference, 70.77–612.7). The PYY_3–36_ + GLP-1_7–36 amide_ combination also significantly increased AIRg compared with vehicle, similar to GLP-1_7–36 amide_ alone (*P* < .05: mean difference, 275.4 mU · L^−1^ · min; 95% CI for difference, 4.48–546.4). Comparison of AIRg in GLP-1_7–36 amide_ alone vs PYY_3–36_ + GLP-1_7–36 amide_ showed no significant difference (mean difference, −66.29 mU · L^−1^ · min; 95% CI for difference, −337.2- 204.7).

No significant differences in S_I_ index were discerned between infusion arms (*P* = .79; Figure 4B). There was no significant difference in mean DI between infusion arms (*P* = .07; Figure 4C).

## Discussion

In this study, we measured the changes in first-phase insulin secretion and S_I_ in response to an acute infusion of PYY_3–36_ and GLP-1_7–36 amide_ in healthy, overweight, nondiabetic humans using an FSIVGTT. As expected from its known action as an incretin hormone, GLP-1_7–36 amide_ infusion significantly increased first-phase insulin secretion in response to the iv glucose compared with vehicle. Similarly, we noted a significant elevation in AIRg with combination PYY_3–36_ + GLP-1_7–36 amide_ infusion. PYY_3–36_ infusion alone resulted in a slight, nonsignificant increase in AIRg compared with vehicle. There seems to be no additive or synergistic effect between PYY_3–36_ and GLP-1_7–36 amide_ on insulin secretion because the combination caused an elevation in AIRg of similar magnitude to GLP-1_7–36 amide_ alone. Furthermore, neither hormone had any significant acute effect on measures of S_I_ in this cohort, hence the changes in disposition indices mirrored the changes in AIRg across all infusion arms.

We have therefore shown that acute, low-dose administration of PYY_3–36_ to overweight humans has no effect on S_I_ and no significant effect on β cell secretory function. In the study by Sloth et al (22), which did report an insulinotropic effect of exogenously administered PYY_3–36_ at 0.2 pmol/kg/min, the insulin response was examined after an ad libitum lunch, but the PYY_3–36_ group surprisingly ate slightly more than the placebo group, perhaps because the PYY infusion day always followed the placebo day, allowing acclimatization to the study environment and a potential order effect. Thus, the increased insulin response with PYY_3–36_ observed by Sloth et al may be merely a response to an increased energy intake at the meal, and also because of an increase in endogenous incretin secretion. In this study, volunteers were acclimatized to experimental conditions, infusions were given in a random order, and we used a standardized method to examine insulin secretion in response to a fixed iv glucose stimulus. Moreover, the use of an iv glucose stimulus avoids any confounding by endogenous incretin secretion, unlike the meal stimulus employed by Sloth et al.

Nevertheless, a limitation of our study is that it does not entirely exclude a modest insulinotropic effect of PYY_3–36_, within the setting of the validated FSIVGTT protocol, which incorporated a large glycemic excursion as standard. A second limitation is that we only studied the first-phase insulin response because the FSIVGTT incorporates a transient glucose stimulus and not the sustained hyperglycaemic stimulus necessary to observe the second-phase insulin response (37). It therefore remains possible that the second-phase insulin response is modulated by PYY_3–36_, and this remains to be tested. Third, it should be noted that we studied low doses of PYY_3–36_ and GLP-1, selected on the basis of prior evidence of being just sufficient to affect insulin secretion. It is possible that higher doses of the combination could have effects on S_I_. This could be explored in future studies. A final limitation of note with our study is that it only examined the effects of PYY_3–36_ and GLP-1 in an acute setting. We speculate that longer-term treatment with the combination of PYY_3–36_ and GLP-1 may ultimately improve S_I_ over the hormones given individually through additive reductions in food intake and therefore greater weight loss.

Importantly, we have shown that the combination of GLP-1 and PYY_3–36_ retains the glucose-lowering insulinotropic effect observed with GLP-1, which adds further support to the concept of multiple gut hormone therapy as a treatment for diabetes and obesity. Future studies should focus on measuring the effects of chronic administration of these gut hormones on weight loss, insulin sensitivity and secretion.
